# Hemophagocytic Lymphohistiocytosis: A Case Series

**DOI:** 10.7759/cureus.2545

**Published:** 2018-04-29

**Authors:** Zainab Fatima, Amina Khan, Usman Tariq, Muhammad Saad Sohail

**Affiliations:** 1 Medicine, Shifa International Hospital, Islamabad, Pakistan, Islamabad, PAK; 2 Shifa Tameer E Millat University, Shifa International Hospital, Islamabad, Pakistan, Islamabad, PAK; 3 Research Assistant, Yale University School of Medicine, New Haven, USA; 4 Internal Medicine, Shifa International Hospital, Islamabad, Pakistan, Islamabad, PAK

**Keywords:** hemophagocytic lymphohistiocytosis, hlh, adult, cd68

## Abstract

Hemophagocytic lymphohistiocytosis (HLH) has been recognized as an inflammatory endpoint for a variety of conditions including autoimmune diseases, malignancies and infections. It can be further classified as primary and secondary HLH. Primary HLH is also known as familial HLH. It usually presents in childhood and can be associated with gene mutations. Secondary HLH is also known as acquired HLH and usually presents in adulthood. In comparison to children, it is difficult to diagnose adults with HLH since it occurs with a variety of different diseases and most of the literature on HLH is derived from a pediatric population.

In this case series, we report two cases of HLH that illustrate the difficulty of making this diagnosis and a brief review of the literature on its pathophysiology, clinical presentation, diagnosis, and a subsequent therapeutic approach.

## Introduction

Hemophagocytic lymphohistiocytosis (HLH) (also known as hemophagocytic syndrome) is a clinical entity characterized by a sustained activation of the mononuclear phagocytic system that may result in an extreme hyperinflammatory response [1]. HLH can occur in two forms: genetic and acquired. Genetic HLH is further categorized into a familial form (familial hemophagocytic lymphohistiocytosis [FHLH]) and is associated with immune deficiencies such as Chédiak-Higashi syndrome (CHS), Griscelli syndrome (GS) and X-linked lymphoproliferative syndrome (XLP). In most cases, HLH is the only manifestation of the familial variant. On the other hand, the acquired form occurs sporadically in association with other immune deficiencies. The genetic and acquired forms may be triggered by infections (viral are the most common) or other stimuli [2]. In the pediatric population, HLH is strongly associated with an infection, while hematologic and/or other malignancies have been more commonly found in adults with HLH.

HLH can be rapidly progressive and potentially fatal if left undiagnosed. A high level of suspicion for HLH is required in patients presenting with splenomegaly, an elevation in liver markers, increased inflammatory markers such as serum ferritin and cytopenias.

## Case presentation

Case 1

A 22-year-old male, known case of hepatitis B for one year, developed a low-grade intermittent fever (100°F-101°F) with chills and rigors, associated with nausea and vomiting related to food intake. A week of this predicament was followed by altered mentation, whereby the patient complained of drowsiness as well as a concomitant decrease in urine output. In our emergency room, he was found to be delirious but had a Glasgow coma scale (GCS) of 15/15. Initial assessment revealed a heart rate (HR) of 110/minute, respiratory rate (RR) of 20/minute, a temperature of 101°F and blood pressure (BP) of 150/100 mm Hg. The patient also had scleral icterus. Rest of the physical examination was unremarkable. Initial investigations at the time of the presentation are shown in Table 1.

**Table 1:** Initial investigations at the time of presentation. AST: Aspartate aminotransferase; ALT: Alanine aminotransferase; ALP: Alkaline phosphatase; CRP: C-reactive protein; LDH: Lactate dehydrogenase; GGT: Gamma-glutamyl transferase.

Initial investigation	Value	Reference range
Hemoglobin	9.10 g/dL	13.5–18.0 g/dL
Hematocrit	24.4%	42–52%
Reticulocyte count	1%	Adults and children, 0.5–2.5%
Total leukocyte count (TLC)	14,300 cells/µL (with bicytopenia, neutrophilia, anisopoikilocytosis but no schistocytes)	(4,000–10,500)/µL
Platelet count	95,000/µL	(150,000–400,000)/µL
AST	325 U/L	05–34 U/L
ALT	242 U/L	0.0–55 U/L
ALP	262 U/L	Adults 40–150 U/L
GGT	456 U/L	Male 12–64 U/L; Female 09–36 U/L
Serum albumin	2.41 g/dL	3.5–5.5 g/dL
Total bilirubin	5.27 mg/dL	Adult 0.2–1.2 mg/dL
Direct bilirubin	3.98 mg/dL	0.0–0.5 mg/dL
LDH	3,965 U/L	140–280 U/L
CRP	105 mg/dL	Up to 5.0 mg/L
Serum sodium	133 mEq/L	Adult 136–145 mEq/L
Serum potassium	4.2 mEq/L	3.5–5.1 mEq/L
Serum bicarbonate	15 mEq/L	Adult 22–29 mEq/L
Serum creatinine	9 mg/dL	Male 0.72–1.25 mg/dL; Female 0.57–1.11 mg/dL
Serum blood urea nitrogen	152 mg/dL	Adult male: <50 Years: 8.9–20.6 mg/dL; >50 Years: 8.4–25.7 mg/dL Adult female: <50 Years: 7.0–18.7 mg/dl; >50 years: 9.8–20.1 mg/dL
Serum calcium	4.8 mg/dL	8.5–10.2 mg/dL
Serum phosphorus	10.1 mg/dL	2.5–4.5 mg/dL

**Figure 1:**
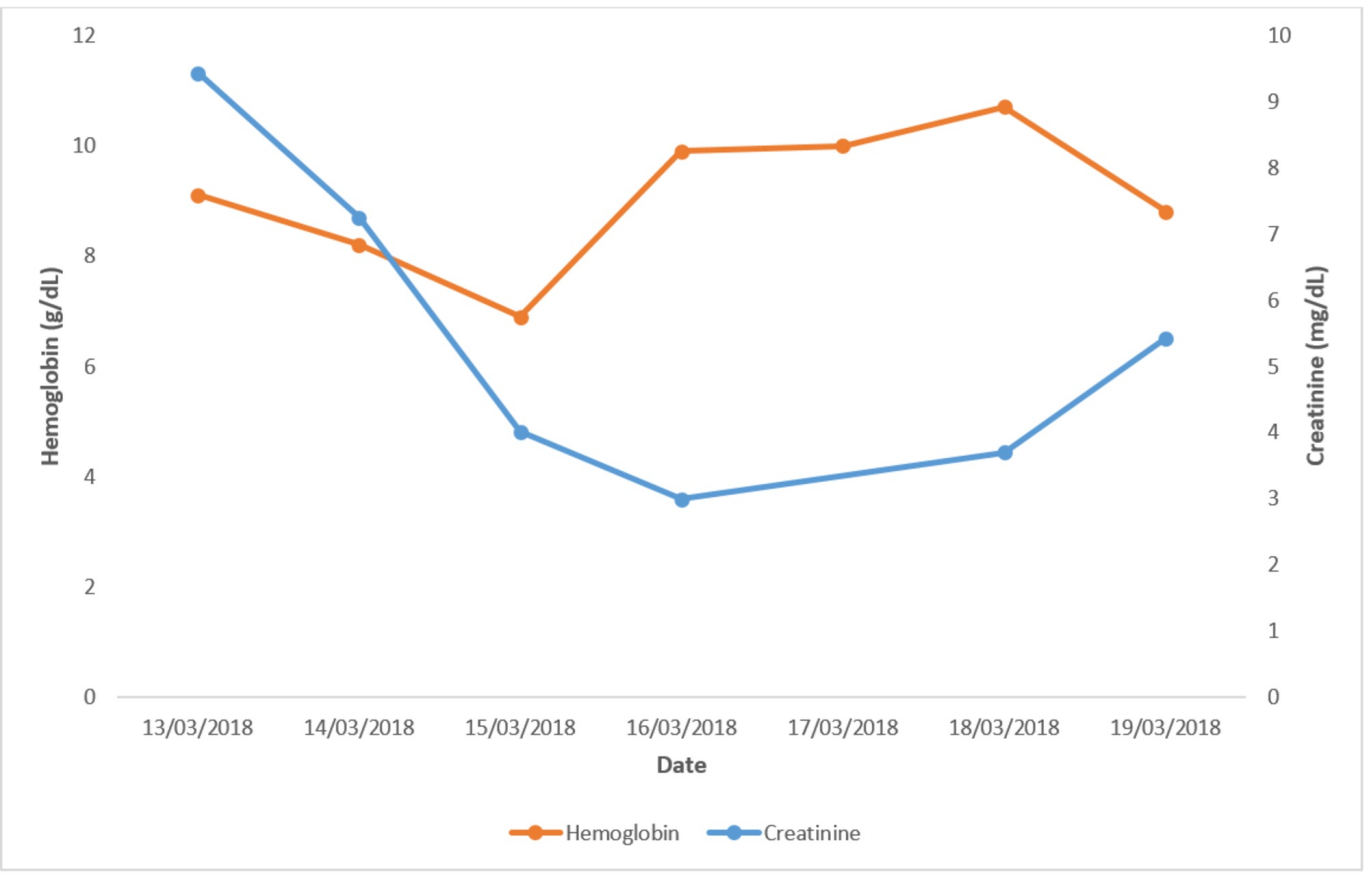
Haemoglobin and creatinine. Graph showing the trend of hemoglobin and creatinine levels over a period of a few days.

**Figure 2:**
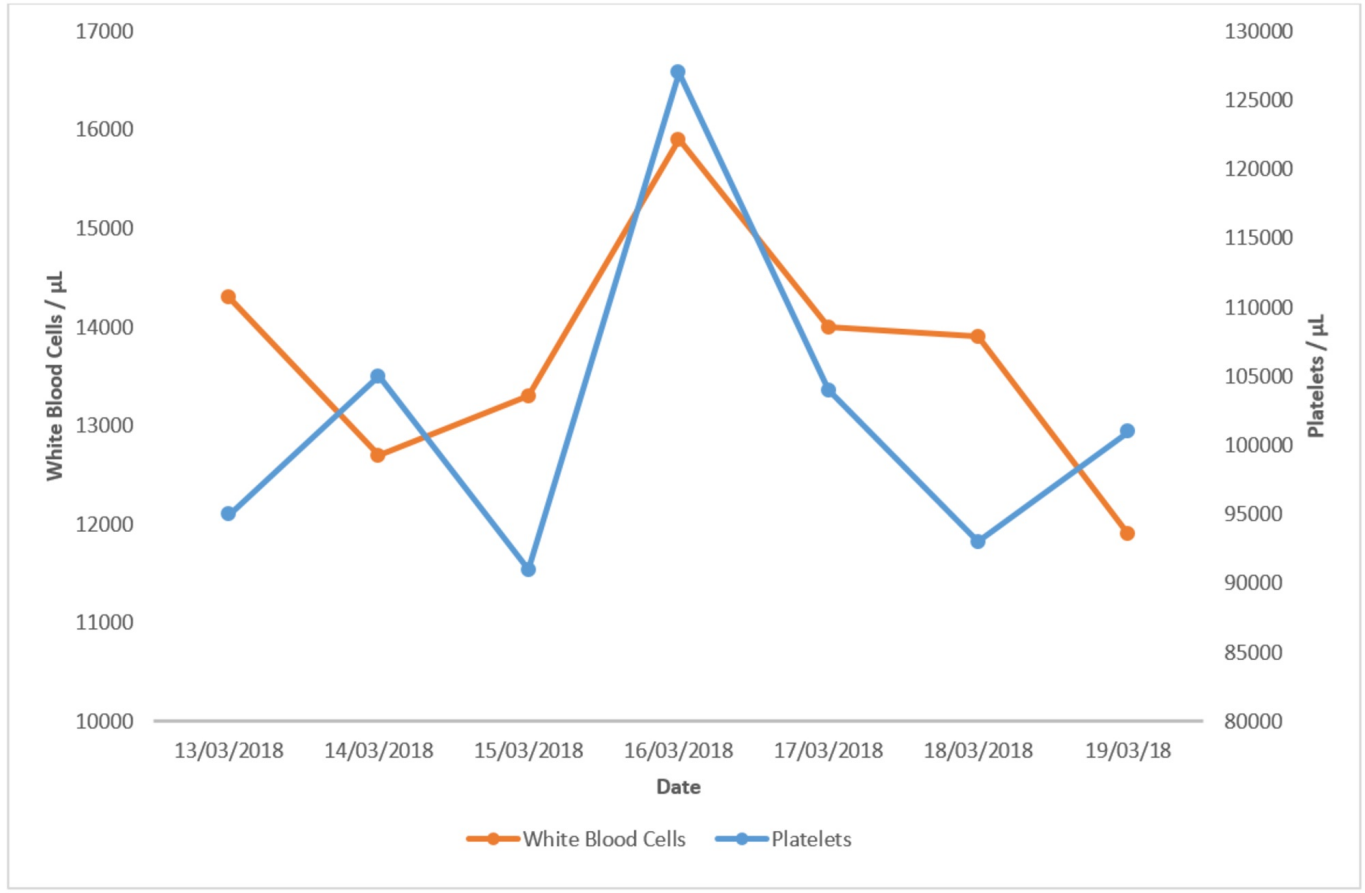
White blood cells and platelets. Graph showing the trend of white blood cells and platelet levels over a period of a few days.

**Figure 3:**
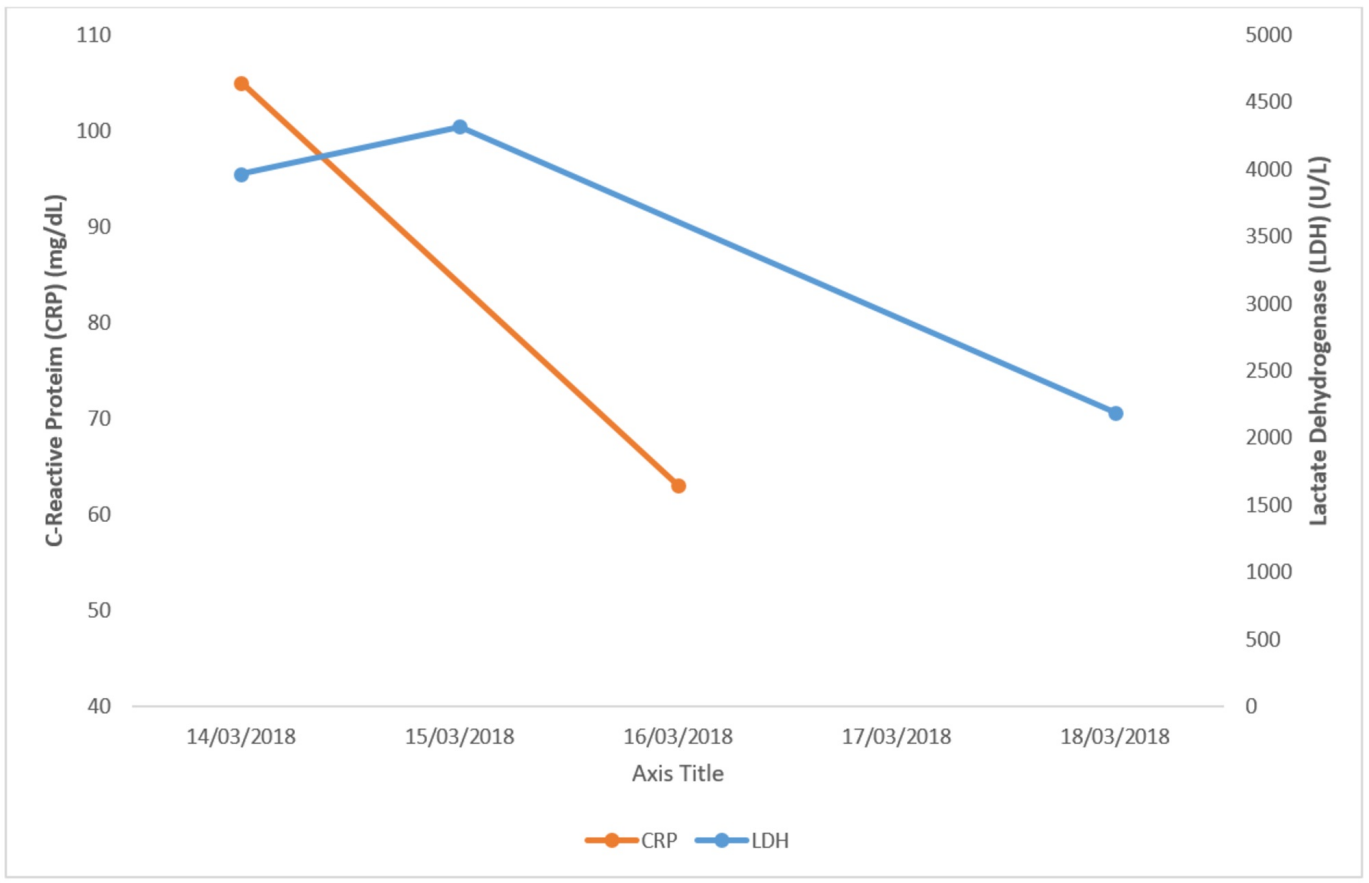
C-reactive protein (CRP) and lactate dehydrogenase (LDH). Graph showing the trend of CRP and LDH over a period of a few days.

The patient was started on broad-spectrum antibiotics (1 gram (g) intravenous vancomycin + 500 milligrams (mg) intravenous imipenem/cilastatin). Figures 1-3 show investigations and their trends over the course of the next few days.

**Figure 4:**
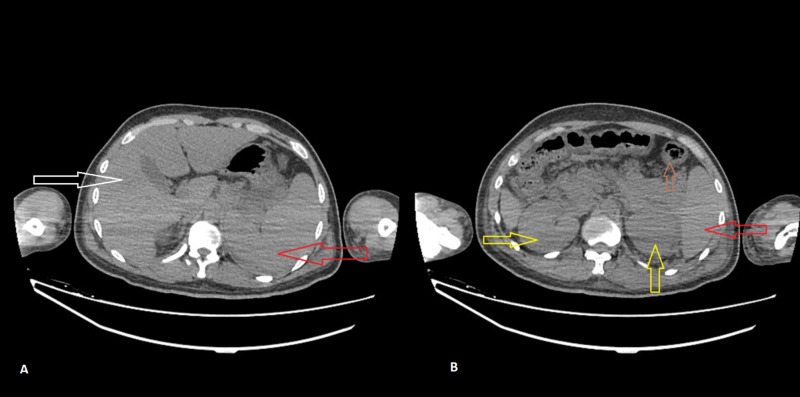
Computed tomography (CT) abdomen without contrast. Image A: White arrow points towards an enlarged liver; red arrow points towards splenomegaly. Image B: Red arrow points towards the inferior portion of the enlarged spleen; yellow arrow points towards swollen and globular appearing kidneys; brown arrow points towards diffuse thickening of the wall of the colon.

Based on his clinical presentation and an elevated serum creatinine, the patient underwent hemodialysis on day two of his admission. A tentative diagnosis of thrombotic thrombocytopenic purpura (TTP) was ruled out based on a low reticulocyte count with a high direct bilirubin value and a positive direct Coombs test. An abdominal ultrasound showed mild abdominopelvic ascites with mild splenomegaly. A head computed tomography (CT) scan was negative for any pathology and CT scan of the chest, abdomen, and pelvis showed bilateral basal consolidation and atelectasis in the lungs, swollen and enlarged pancreas, diffuse thickening of the walls of the ascending, transverse and descending colon, hepatosplenomegaly, swollen and globular appearing kidneys, and intramural hemorrhages with peritoneal hemorrhagic fluid (Figure 4).

**Figure 5:**
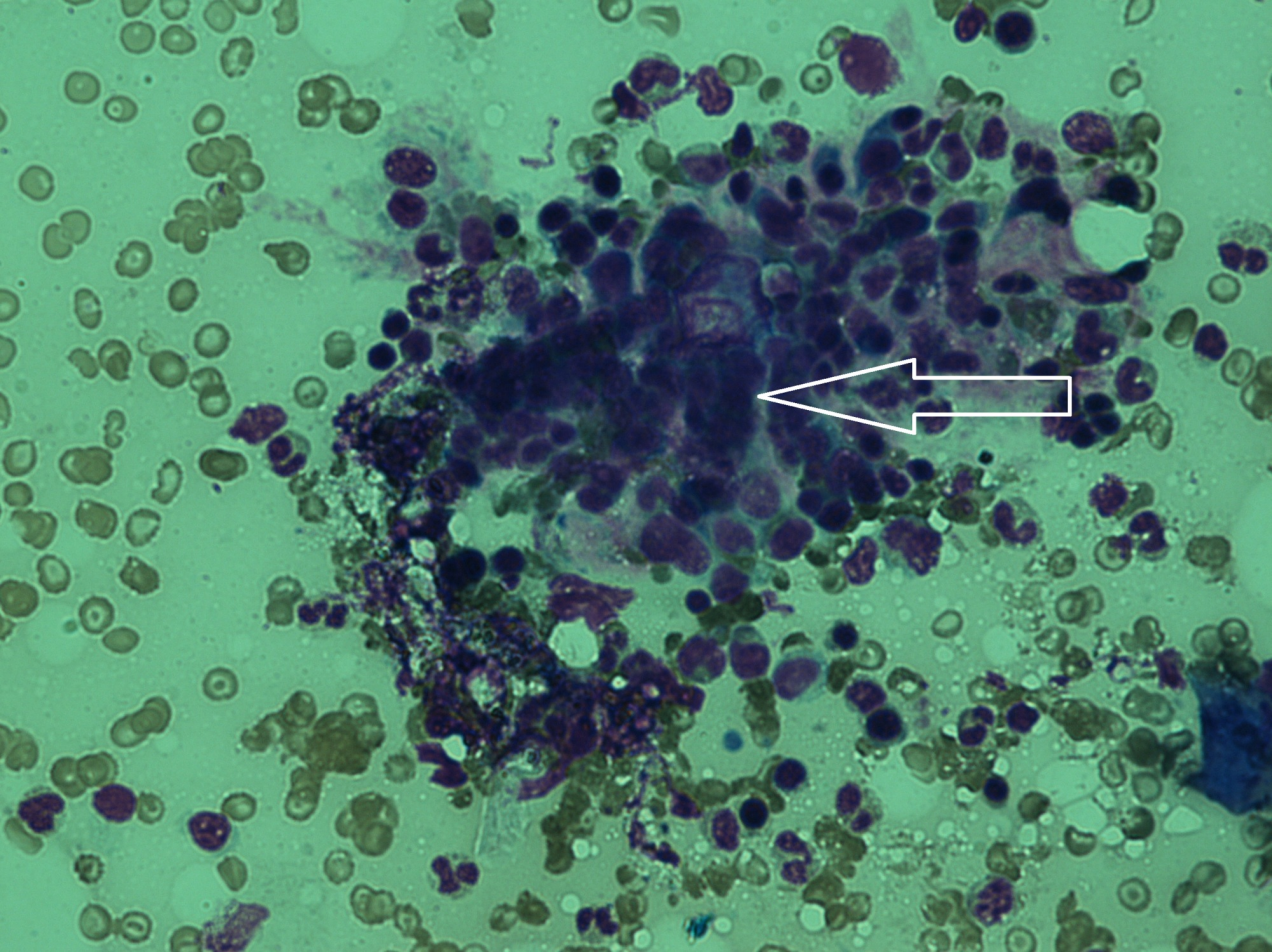
Bone marrow biopsy. White arrow pointing towards hemophagocytic activity on bone marrow biopsy.

**Figure 6:**
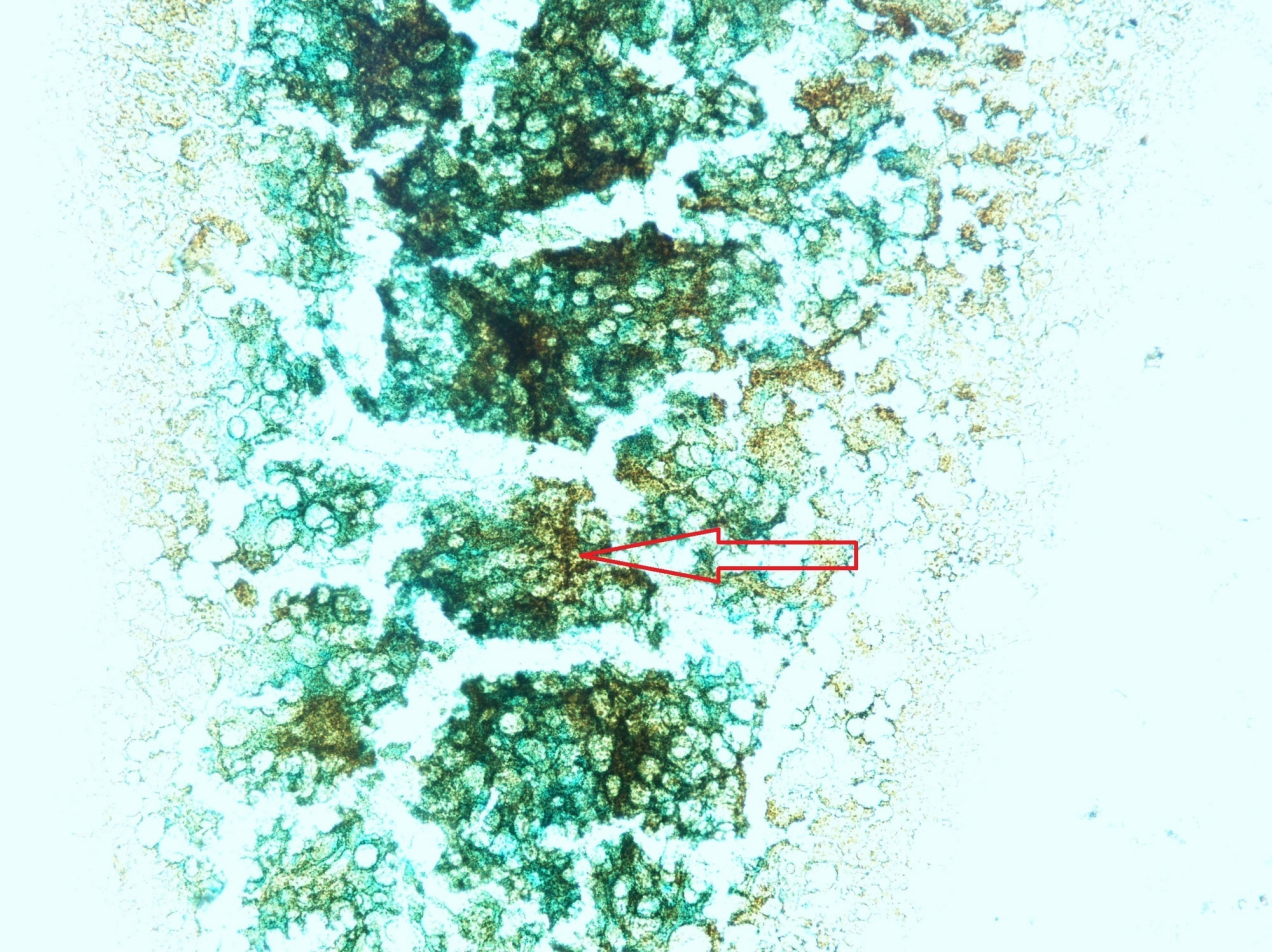
Bone marrow biopsy on Perl's Prussian blue stain. Red arrow pointing towards excessive iron deposits in the bone marrow on Perl's Prussian blue stain.

**Figure 7:**
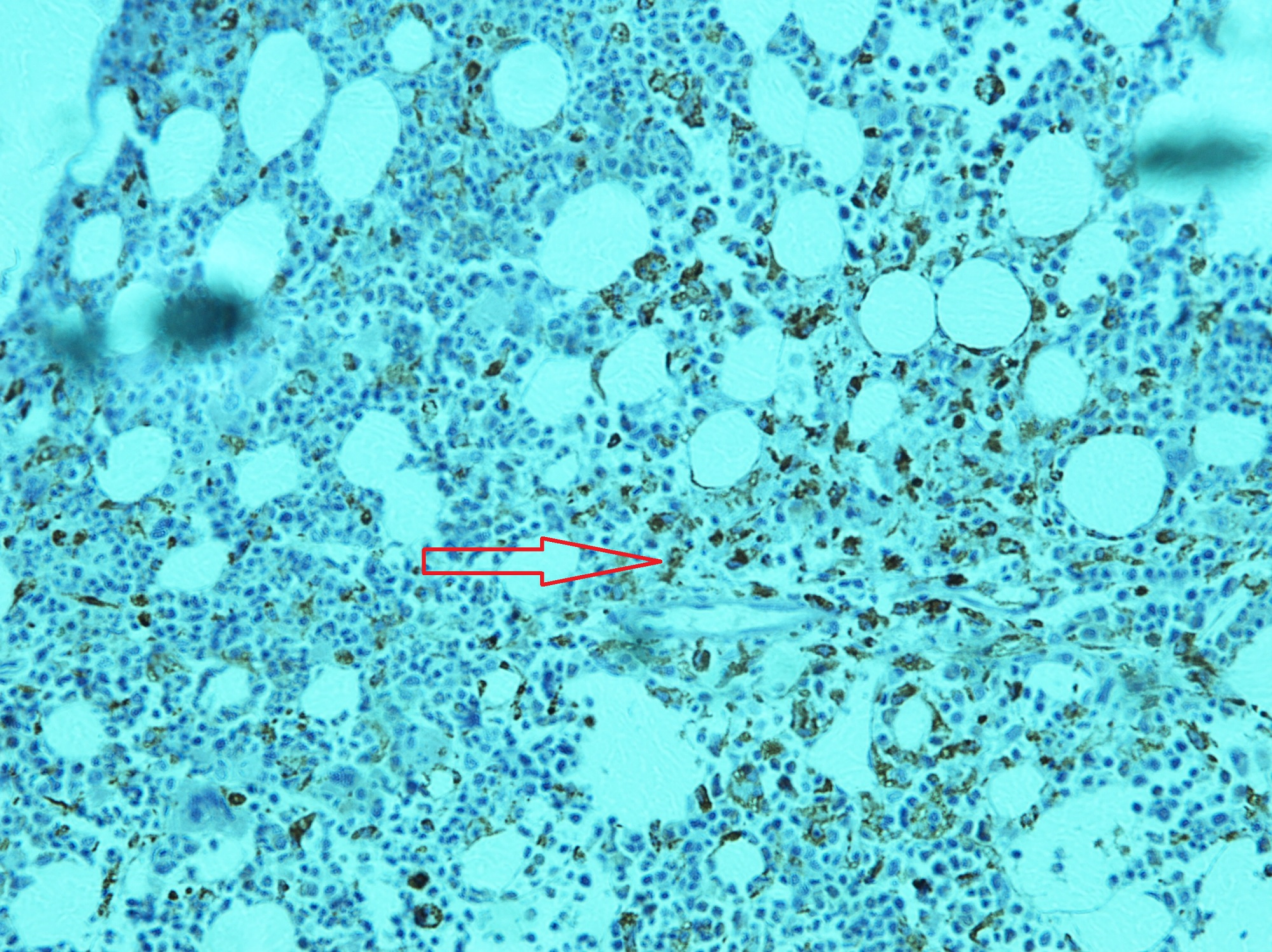
Immunohistochemistry of bone marrow biopsy. Red arrow pointing towards histiocytic activity on CD68.

Additional investigations showed a serum fibrinogen level of 292 mg/dL, serum triglyceride level of 496 mg/dL, and a serum ferritin level of 30,304 ng/mL. C-reactive protein level (CRP) was 105 mg/L while serum lactate dehydrogenase (LDH) was 3,965 U/L. A hepatitis panel blood test was only positive for hepatitis B surface antigen (HBsAg). Antinuclear antibody (ANA) was negative, thus ruling out systemic lupus erythematosus (SLE) and other autoimmune etiologies. Blood cultures assessed after five days were unrevealing for any microbial growth. Considering a lack of clinical improvement, a bone marrow biopsy was performed on the 10th day of admission which showed 80% cellularity with increased hemophagocytic activity. Immunohistochemistry revealed increased histiocytes on CD68, and Iron stain revealed 2+ stainable iron (Figures 5-7).

According to the HLH-2004 trial, this patient fulfilled six features out of the eight-point diagnostic criteria for HLH, including a persistent fever >101 F, bicytopenia, splenomegaly on an abdominal CT scan, hypertriglyceridemia and/or hypofibrinogenemia, hyperferritinemia and a histiocytic activity on a bone marrow aspirate. In this case, the underlying cause of HLH was suspected to be a chronic hepatitis B infection. He was started on etoposide (100 mg/m²) along with dexamethasone as per the HLH treatment protocol.

Case 2

A 44-year-old male presented to our outpatient department with complaints of a continuous fever (100°F-102°F), loss of appetite, undocumented weight loss and fatigue for two weeks. The fever was not associated with chills and responded to acetaminophen. He also complained of occasional gum bleeds. A review of systems was otherwise unremarkable. Initial assessment revealed a heart rate of 110/minute, respiratory rate of 17/minute, a temperature of 102°F and blood pressure of 140/90 mm Hg. The rest of the physical exam was unremarkable. Initial investigations at the time of presentation are shown in Table 2.

**Table 2:** Initial investigations of case 2 patient at the time of presentation. AST: Aspartate aminotransferase; ALT: Alanine aminotransferase; ALP: Alkaline phosphatase; LDH: Lactate dehydrogenase; GGT: Gamma-glutamyl transferase.

Initial investigation	Value	Reference range
Haemoglobin	13 g/dL	13.5–18.0 g/dL
Hematocrit	37.5%	42–52%
Reticulocyte count	3.5%	Adults and children 0.5–2.5%
Total leukocyte count (TLC)	3,500 cells/µL	(4,000–10,500)/µL
Platelet count	101,000/µL	(150,000–400,000)/µL
AST	205 U/L	05–34 U/L
ALT	147 U/L	0.0–55 U/L
ALP	295 U/L	Adults 40–150 U/L
GGT	213 U/L	Male 12–64 U/L; Female 09–36 U/L
Total bilirubin	1.71 mg/dL	Adult 0.2–1.2 mg/dL
Direct bilirubin	0.96 mg/dL	0.0–0.5 mg/dL
LDH	485 U/L	140–280 U/L
Serum sodium	132 mEq/L	Adult 136–145 mEq/L
Serum potassium	3.74 mEq/L	3.5–5.1 mEq/L
Serum bicarbonate	26 mEq/L	Adult 22–29 mEq/L
Serum creatinine	0.61 mEq/L	Male 0.72–1.25 mg/dL; Female 0.57–1.11 mg/dL
Serum blood urea nitrogen	14 mg/dL	Adult male: <50 Years: 8.9–20.6 mg/dL; >50 Years: 8.4–25.7 mg/dl Adult female: <50 Years: 7.0–18.7 mg/dl; >50 years: 9.8–20.1 mg/dl

**Figure 8:**
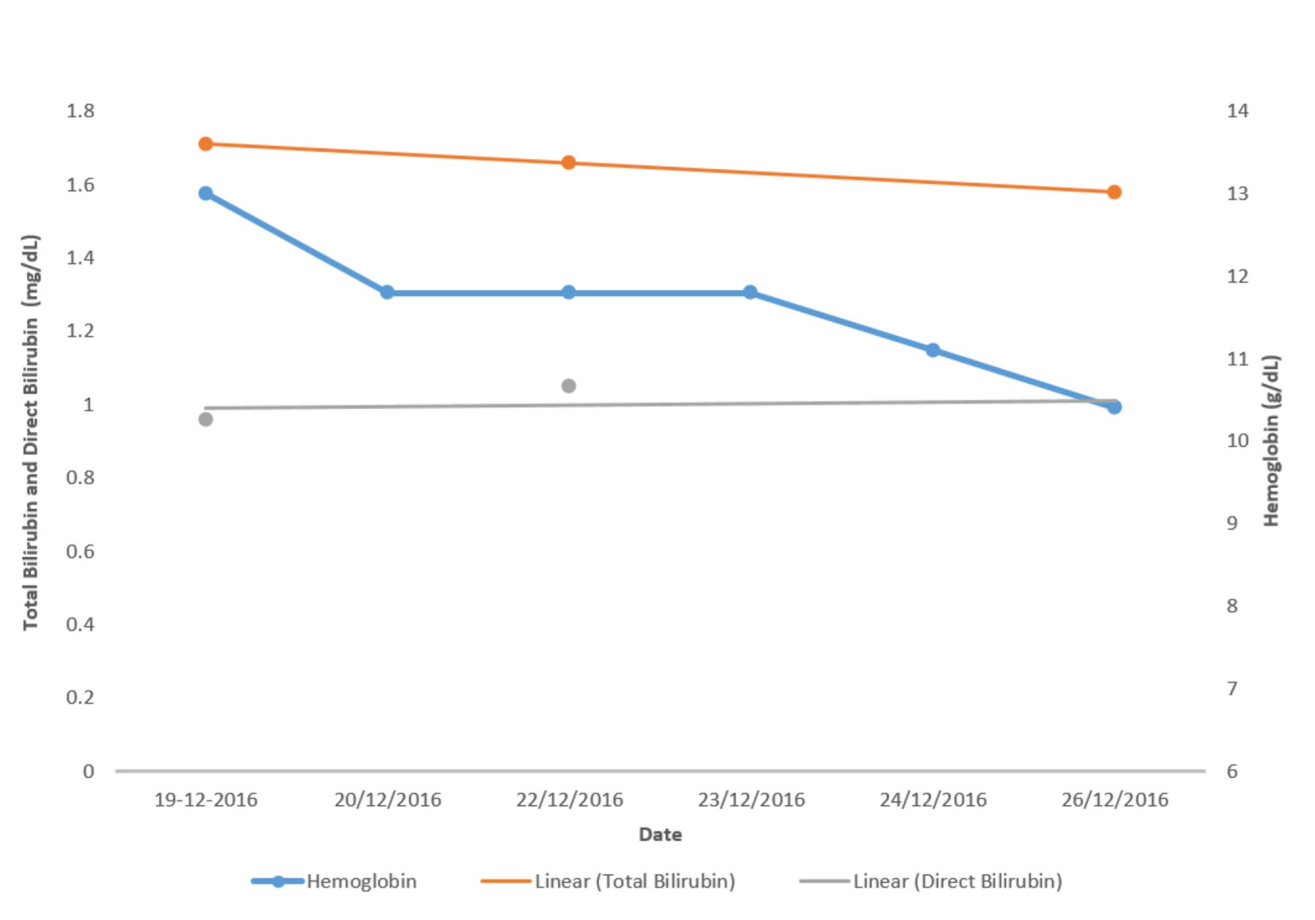
Haemoglobin, bilirubin and direct bilirubin. Graph showing the trend of hemoglobin, total bilirubin and direct bilirubin levels over a period of a few days.

**Figure 9:**
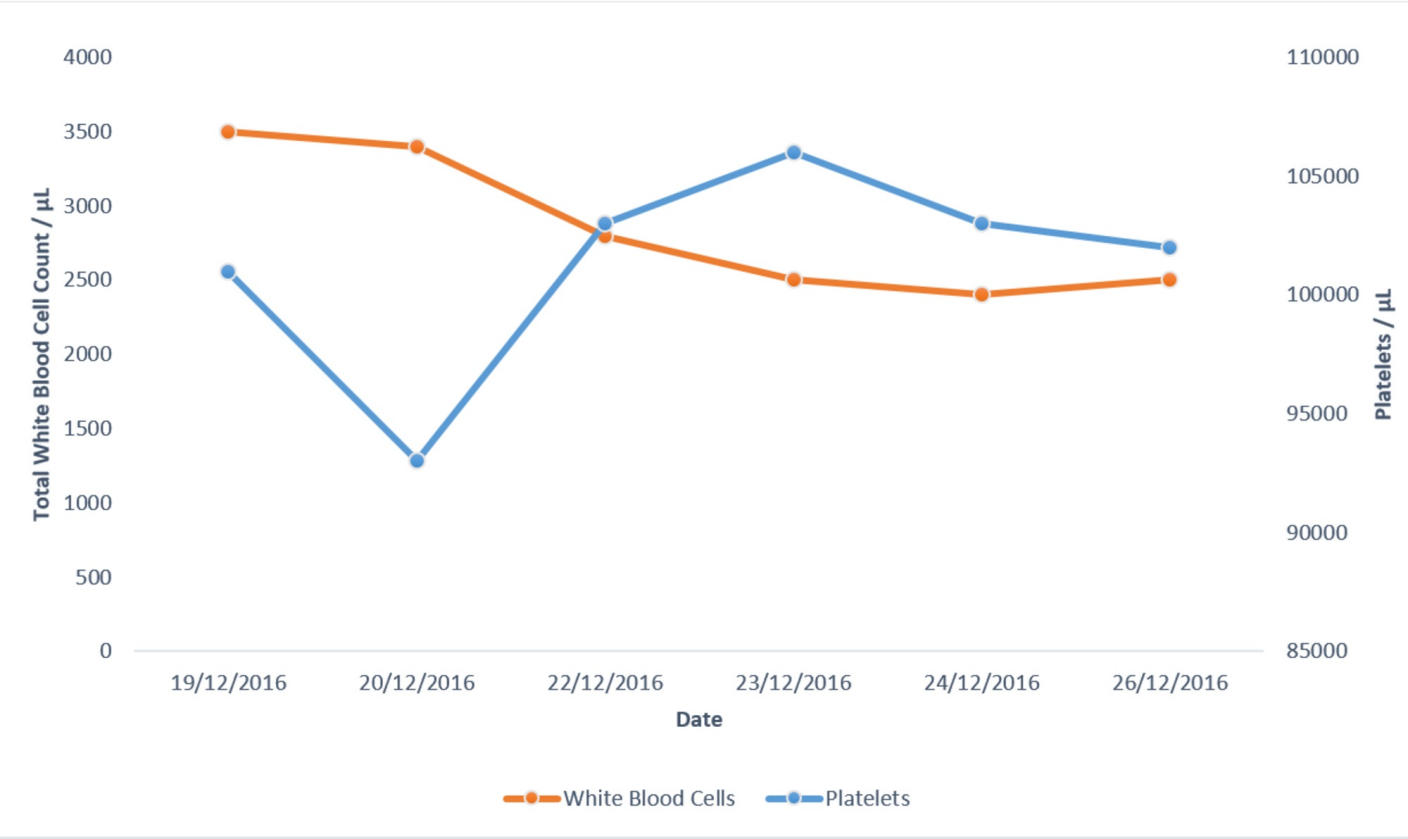
White blood cells and platelets. Graph showing the trend of white blood cells and serum platelet levels over a period of a few days.

**Figure 10:**
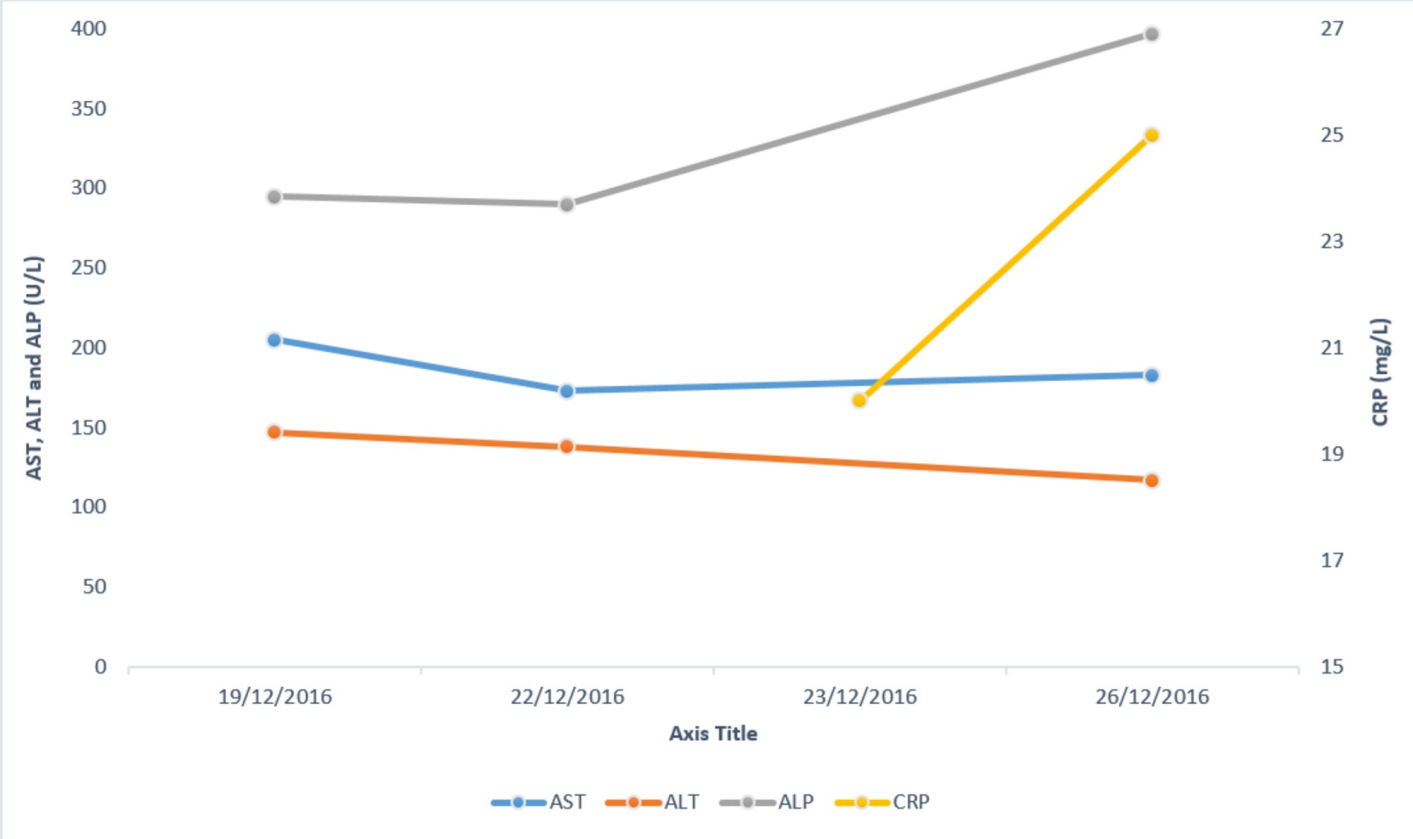
AST, ALP, ALT and CRP. Graph showing the trend of AST, ALT, ALP and CRP levels over a period of a few days. AST: Aspartate aminotransferase; ALT: Alanine aminotransferase; ALP: Alkaline phosphatase; CRP: C-reactive protein.

The patient was then started on a combination of 1 g intravenous vancomycin and 500 mg intravenous imipenem/cilastatin. Figures 8-10 show the investigation results and their trends over the course of the next few days while the patient was admitted to our setting.

**Figure 11:**
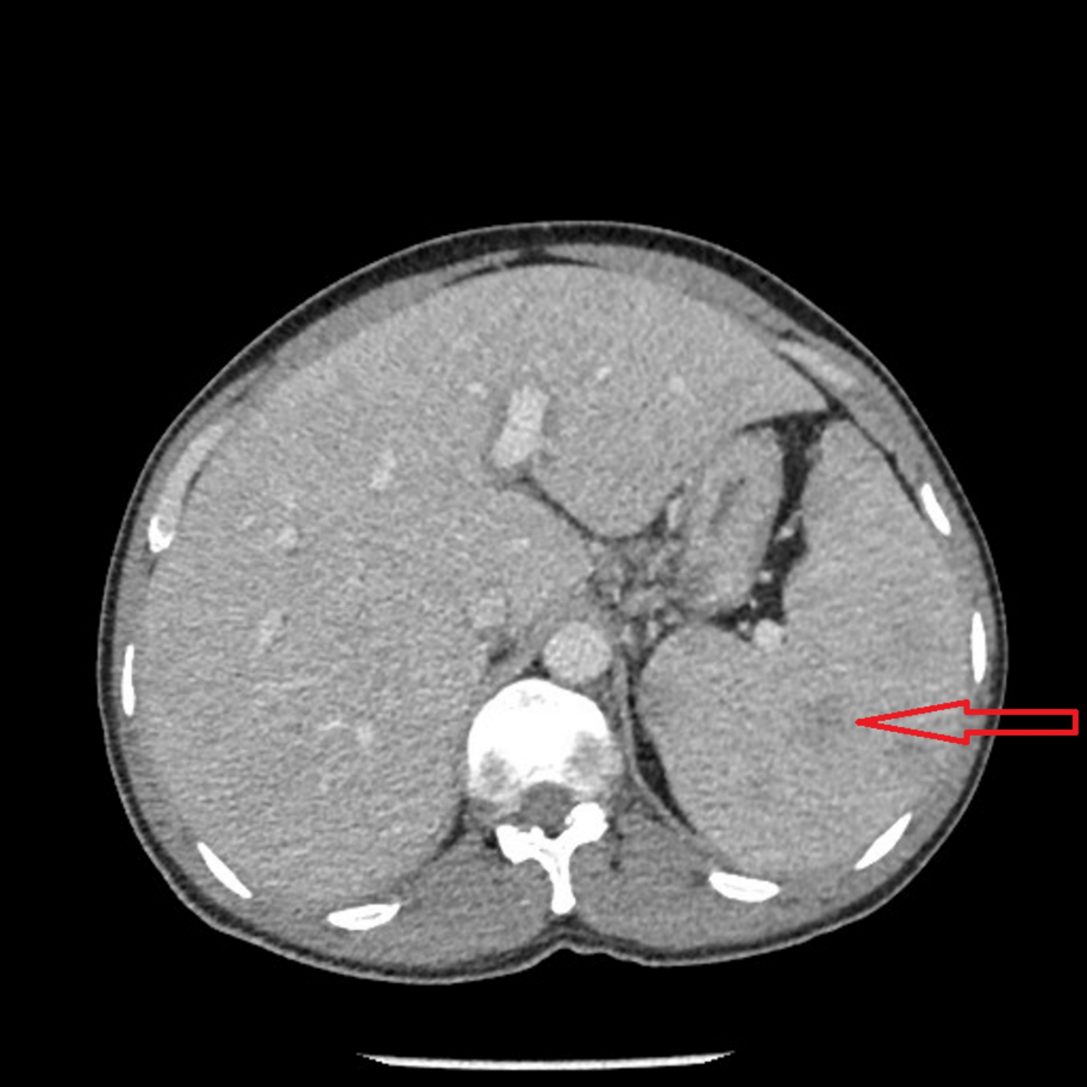
CT abdomen with contrast. CT abdomen with contrast reveals moderate hepatosplenomegaly with the red arrow pointing towards hypodense lesions in the spleen. CT: Computed tomography

An abdominal CT scan showed moderate hepatosplenomegaly with multiple hypodense lesions in the spleen. There was a mild enlargement of the left inguinal, paracardiac and subdiaphragmatic lymph nodes. Diffuse omento-mesenteric congestion and pelvic ascites were also noted (Figure 11).

Despite broad-spectrum antibiotic therapy, the patient remained febrile. Additional investigations showed a serum fibrinogen level of 132 mg/dL, serum triglycerides level of 257 mg/dL, serum ferritin was 5,092 ng/mL, serum LDH was 4,85 U/L and CRP was 20 mg/dL. Prothrombin time/international normalized ratio (PT/INR), serum vitamin B12, and serum folic acid levels were normal. A suspicion of autoimmune hepatitis was ruled out based on a negative anti-liver-kidney microsomal antibody (anti-LKM-1) test. Viral serologies revealed that the patient was negative for cytomegalovirus (CMV), herpes simplex virus (HSV), hepatitis B virus and hepatitis C virus. Blood cultures assessed after five days were negative.

Considering a lack of clinical improvement, a bone marrow biopsy was performed on the sixth day of admission which revealed normocellular marrow with an increase in dysplastic megakaryocytes. Hyperplastic erythropoiesis with nuclear cytoplasm asynchrony was also noted. Moderate hemophagocytic activity was evident with prominent histiocytes and plasma cells. Based on his abdominal CT report, he later underwent a left inguinal lymph node biopsy on the seventh day of his admission, which revealed reactive hyperplasia but was negative for malignancy.

According to the HLH-2004 trial, this patient fulfilled six out of the eight-point diagnostic criteria for HLH including a fever of >101°F, bicytopenia, splenomegaly on abdominal CT scan, hypertriglyceridemia and/or hypofibrinogenemia, hyperferritinemia and a histiocytic activity on a bone marrow aspirate. However, the underlying etiology remained unclear. He was started on etoposide (100 mg/m²) along with dexamethasone as per the HLH treatment protocol. After completing eight sessions of etoposide, the patient was admitted again with complaints of abdominal pain. Hepatomegaly was noted and liver enzymes were found to be elevated, which prompted a liver biopsy that revealed a T-cell rich, histiocytic B-cell lymphoma. The patient was subsequently started on chemotherapy with rituximab, cyclophosphamide, vincristine, prednisone, and doxorubicin. A good response to the treatment was noted, and a complete remission was ultimately achieved on positron emission tomography (PET)/CT scans.

## Discussion

Types of HLH and their associated etiologies

HLH has been recognized as an inflammatory endpoint for a variety of conditions including autoimmune diseases, malignancies, and infections. It can be classified as primary and secondary. Primary HLH (also known as familial HLH) usually presents in childhood. It is associated with mutations in genes such as MUNC 13-4, perforin 1 (PRF1), Syntaxin-122 and Syntaxin-binding protein which can affect the cytotoxic function of T-lymphocytes and natural killer (NK) cells. Although FHLH can occur sporadically with these mutations, it usually presents after a viral infection, with Epstein–Barr Virus (EBV) as the most likely culprit [3-4].

Secondary HLH is also known as acquired HLH and commonly presents in adulthood. It is usually associated with an underlying infection, malignancy or an autoimmune disease which can lead to a hyperactive immune response [1,3]. There is a growing body of evidence that suggests that patients with secondary HLH may also have a genetic predisposition [4]. Occasionally, HLH may occur without a known underlying condition, referred to as idiopathic HLH. However, it is vital to differentiate between primary and secondary HLH because treatment of an underlying condition may prove to be beneficial in the latter.

A retrospective cohort study performed by Otrock and Eby on 73 adults with secondary HLH showed that infection was the most common underlying etiology (41.1%), followed by malignancy (28.8%), autoimmune diseases (6.8%), and post solid organ transplant (2.7%). However, no discernable cause could be elucidated in a large subset (17.8%) of the documented cases. EBV and human immunodeficiency virus (HIV) were the most frequent infections causing HLH, while lymphoma was the most commonly associated malignancy [1]. Other viral etiologies implicated in HLH development include hepatitis B (HBsAg was positive in our patient), hepatitis C and CMV [5]. A case series submitted from China showed similar results, indicating malignancy and viral infections as the most common factors for secondary HLH [6].

While a number of cases of lymphoma-associated HLH have been reported, there is limited literature on HLH secondary to hepatitis B infections. In the study performed by Otrock and Eby, only one patient had hepatitis B-associated HLH [1].

Clinical presentation and diagnosis

The clinical features of HLH can be non-specific, which adds to the difficulty of its diagnosis. This is evident from a Chinese study consisting of 103 adults which concluded that >96% of patients with HLH presented with a high-grade fever, while 79.6% had splenomegaly, and 53.4% had lymphadenopathy. Among the laboratory findings, 98.4% of the patients had a significant elevation of serum ferritin (≥500 µg/L), cytopenia was found in 98% of the patients, bone marrow hemophagocytosis was seen in 87.4% of the patients and hypertriglyceridemia was noted in 85% of the patients [6].

The pathophysiology of the clinical presentation and associated laboratory findings in HLH can be explained by the mechanics of a hyperactive immune response. Several cell types are involved in the pathophysiology of HLH, including macrophages, NK-cells, and cytotoxic T-lymphocytes. Macrophages typically serve as antigen presenting cells to present foreign antigens to lymphocytes for either direct destruction or antibody development. In various forms of HLH, impaired cytotoxic function leads to an uncontrolled inflammatory response with the activation and expansion of interferon-γ (IFN-γ)-producing T-cells. High levels of IFN-γ lead to macrophage activation and overproduction of proinflammatory cytokines. Cytokines, in turn, can cause organ damage when excreted in excessive amounts. NK-cells directly destroy damaged or infected cells, independent of the major histocompatibility complex (MHC). Cytotoxic T-lymphocytes, while similar to NK-cells, kill autologous cells carrying foreign antigens associated with MHC Class I. An elevation in tumor necrosis factor-alpha (TNF-α) and INF-γ explains the cytopenia. Elevated cytokines also lead to an increase in body temperature and precipitate fever. Splenomegaly occurs due to direct infiltration by macrophages. Increase TNF-α levels can decrease lipoprotein lipase levels which can lead to hypertriglyceridemia [4-5].

A diagnosis of HLH, as determined by the Histiocyte Society in 2004, is based on the following criteria [7]:

Molecular diagnosis confirming HLH, and/or five of the following parameters:

• Fever >38.5°C (>101.3°F)

• Splenomegaly

• Cytopenias (at least two cell lines are affected with adult hemoglobin <10 g/dL, platelets <10,000/µL and absolute neutrophil count <1000/µL)

• Hyperferritinemia with serum ferritin >500 µg/L

• Hypertriglyceridemia (with triglycerides levels >265 mg/dL) and/or hypofibrinogenemia (fibrinogen levels <1.5 g/L)

• Hemophagocytosis in the bone marrow/lymph nodes/spleen

• Low or absent NK cell activity

• An increase in soluble CD25 (increase in soluble Il-2 receptor) >2,400 U/mL

Other common signs consistent with the diagnosis include hyponatremia, edema, rash, hypoalbuminemia, elevated LDH, and other lipid abnormalities [7].

A major obstacle in diagnosing a patient with HLH is the overlap of symptoms with a variety of other illness such as sepsis, primary liver failure, multiple organ dysfunction syndrome, and TTP. It is especially challenging to differentiate between sepsis and HLH and there exists no ideal test to distinguish between the two illnesses. However, sepsis is usually caused by bacterial, viral or fungal infections which were unlikely in our patients since blood cultures were negative. Secondly, patients with HLH tend to have a rising trend in serum ferritin levels, while patients with sepsis usually have an elevated, but static value for serum ferritin [8].

Liver failure can be differentiated from HLH by the multi-organ involvement in the latter. It is possible that a patient with HLH could present with jaundice and deranged liver function tests (like our patient in case 1) and thus mimic acute liver failure, which could lead to a delay in making the correct diagnosis, especially if clinicians have a low suspicion for HLH. As far as TTP is concerned, anemia in TTP is microangiopathic (with schistocytes on peripheral smear and a negative Coombs test). Multi-organ dysfunction syndrome (MODS), like HLH, can involve multiple organs; however, an elevated serum ferritin level is more indicative of HLH than MODS [8].

Treatment

Hematopoietic stem cell transplantation (HSCT) was first described by Fischer et al. as a cure for HLH. Since then, it has become the gold standard for treating pediatric HLH as well as recurrent HLH in adults [4].

The objective of the pharmacological treatment of HLH is to control the hyperactive immune system. HLH associated with viral infections is treated according to the HLH-2004 protocol, which includes etoposide (a standard dose of 150 mg/m²) with dexamethasone [4,7]. This treatment was given to our first patient, who responded well and is currently completing his regimen. Our second patient was given the same treatment but was later diagnosed with a T-cell rich B-cell lymphoma. He was then treated with rituximab plus cyclophosphamide, vincristine, doxorubicin, and prednisone. He is currently in remission.

It should also be noted that HLH-2004 protocol only caters to the pediatric population. Currently, no treatment protocol exists for adults affected with HLH [4].

## Conclusions

Considering the wide array of clinical presentations of HLH, supplemented with the fact that there exists a very limited amount of literature on the incidence of HLH in Pakistan, we highlight the possibility that the disease might be underdiagnosed in our region. This advocates the need for further studies to ascertain the prevalence of the disease and its variants. The patients in our setup presented with nonspecific signs and symptoms and had differing etiologies for their ultimate diagnosis, which underscores the challenge of diagnosing HLH. Therefore, a need for high clinical suspicion cannot be overemphasized. We treated our patients based on the HLH protocol and have found a favorable clinical outcome to date.
